# Supplementary material for: Empirical methods for controlling false positives and estimating confidence in ChIP-Seq peaks
Source: BMC Bioinformatics. 2008 Dec 5;9:523. doi: 10.1186/1471-2105-9-523 (PMC2628906; doi:10.1186/1471-2105-9-523)
Supplement: Additional File 1 — A variety of html documents from the USeq web site detailing the available applications, their best usage, output file type descriptions, command line menus, etc. [file 1471-2105-9-523-S1.zip › USeqUserGuides/JavaDoc.zip!/JavaDoc/util/bio/parsers/UCSCGeneLine.html]

UCSCGeneLine


|  |  |  |
| --- | --- | --- |
| **Overview** \| **Package** \| **Class** \| **Use** \| **Tree** \| **Deprecated** \| **Index** \| **Help** | |  |
| **PREV CLASS**   **NEXT CLASS** | **FRAMES**    **NO FRAMES**     **All Classes** |  |
| SUMMARY: NESTED \| FIELD \| CONSTR \| METHOD | DETAIL: FIELD \| CONSTR \| METHOD |  |


---


## util.bio.parsers Class UCSCGeneLine

```
java.lang.Object
  util.bio.parsers.UCSCGeneLine
```

---

``` public class UCSCGeneLine extends java.lang.Object ```

---

**Constructor Summary**

`UCSCGeneLine(java.lang.String[] tokens,
int numToSubtractFromEnd,
boolean displayNamePresent)`

`UCSCGeneLine(java.lang.String name,
Coordinate corr)`


| **Method Summary** | |
| --- | --- |
| `int` | `getCdsEnd()` |
| `int` | `getCdsStart()` |
| `java.lang.String` | `getChrom()` |
| `java.lang.String` | `getDisplayName()` |
| `ExonIntron[]` | `getExons()` |
| `java.lang.String` | `getName()` |
| `java.lang.String` | `getNotes()` |
| `float[]` | `getScores()` |
| `java.lang.String` | `getStrand()` |
| `int` | `getTss()` |
| `int` | `getTxEnd()` |
| `int` | `getTxStart()` |
| `java.lang.String` | `scoresToString()` |
| `void` | `setCdsEnd(int cdsEnd)` |
| `void` | `setCdsStart(int cdsStart)` |
| `void` | `setDisplayName(java.lang.String displayName)` |
| `void` | `setExons(ExonIntron[] exons)` |
| `void` | `setName(java.lang.String name)` |
| `void` | `setNotes(java.lang.String notes)` |
| `void` | `setScores(float[] scores)` |
| `void` | `setTss(int tss)` |
| `void` | `setTxEnd(int txEnd)` |
| `void` | `setTxStart(int txStart)` |
| `java.lang.String` | `simpleToString()` |
| `java.lang.String` | `toString()` |

**Methods inherited from class java.lang.Object**

`equals, getClass, hashCode, notify, notifyAll, wait, wait, wait`

**Constructor Detail**

### UCSCGeneLine

```
public UCSCGeneLine(java.lang.String name,
                    Coordinate corr)
```

---


### UCSCGeneLine

```
public UCSCGeneLine(java.lang.String[] tokens,
                    int numToSubtractFromEnd,
                    boolean displayNamePresent)
```


**Method Detail**

### scoresToString

```
public java.lang.String scoresToString()
```

---


### simpleToString

```
public java.lang.String simpleToString()
```

---


### toString

```
public java.lang.String toString()
```

:   **Overrides:**: `toString` in class `java.lang.Object`

---


### getCdsEnd

```
public int getCdsEnd()
```

---


### getCdsStart

```
public int getCdsStart()
```

---


### getChrom

```
public java.lang.String getChrom()
```

---


### getExons

```
public ExonIntron[] getExons()
```

---


### getName

```
public java.lang.String getName()
```

---


### getStrand

```
public java.lang.String getStrand()
```

---


### getTxEnd

```
public int getTxEnd()
```

---


### getTxStart

```
public int getTxStart()
```

---


### getNotes

```
public java.lang.String getNotes()
```

---


### setNotes

```
public void setNotes(java.lang.String notes)
```

---


### setName

```
public void setName(java.lang.String name)
```

---


### getTss

```
public int getTss()
```

---


### getScores

```
public float[] getScores()
```

---


### setScores

```
public void setScores(float[] scores)
```

---


### getDisplayName

```
public java.lang.String getDisplayName()
```

---


### setDisplayName

```
public void setDisplayName(java.lang.String displayName)
```

---


### setCdsEnd

```
public void setCdsEnd(int cdsEnd)
```

---


### setCdsStart

```
public void setCdsStart(int cdsStart)
```

---


### setExons

```
public void setExons(ExonIntron[] exons)
```

---


### setTss

```
public void setTss(int tss)
```

---


### setTxEnd

```
public void setTxEnd(int txEnd)
```

---


### setTxStart

```
public void setTxStart(int txStart)
```


---


|  |  |  |
| --- | --- | --- |
| **Overview** \| **Package** \| **Class** \| **Use** \| **Tree** \| **Deprecated** \| **Index** \| **Help** | |  |
| **PREV CLASS**   **NEXT CLASS** | **FRAMES**    **NO FRAMES**     **All Classes** |  |
| SUMMARY: NESTED \| FIELD \| CONSTR \| METHOD | DETAIL: FIELD \| CONSTR \| METHOD |  |


---
